# Temporal Transition of Mechanical Characteristics of HUVEC/MSC Spheroids Using a Microfluidic Chip with Force Sensor Probes

**DOI:** 10.3390/mi7120221

**Published:** 2016-12-05

**Authors:** Keitaro Ito, Shinya Sakuma, Masaki Kimura, Takanori Takebe, Makoto Kaneko, Fumihito Arai

**Affiliations:** 1 Department of Micro-Nano Systems Engineering, Nagoya University, Nagoya 464-8603, Aichi, Japan; sakuma@mech.nagoya-u.ac.jp (S.S.); arai@mech.nagoya-u.ac.jp (F.A.); 2 Division of Gastroenterology, Hepatology and Nutrition, Cincinnati Children’s Hospital Medical Center, Cincinnati, OH 45229-3039, USA; Masaki.Kimura@cchmc.org (M.K.); Takanori.Takebe@cchmc.org (T.T.); 3 Department of Regenerative Medicine, Yokohama City University, Yokohama 236-0004, Kanagawa, Japan; 4 Department of Mechanical Engineering, Osaka University, Suita 565-0871, Osaka, Japan; mk@mech.eng.osaka-u.ac.jp

**Keywords:** mechanical characterization, microfluidic chip, spheroid, force sensor probe

## Abstract

In this paper, we focus on the mechanical characterization of co-cultured spheroids of human umbilical vein endothelial cells (HUVECs) and mesenchymal stem cells (MSC) (HUVEC/MSC spheroids). HUVEC/MSC spheroids aggregate during culture, thereby decreasing in size. Since this size decrease can be caused by the contractility generated by the actomyosin of MSCs, which are intracellular frames, we can expect that there is a temporal transition for the mechanical characteristics, such as stiffness, during culture. To measure the mechanical characteristics, we use a microfluidic chip that is integrated with force sensor probes. We show the details of the measurement configuration and the results of mechanical characterization of the HUVEC/MSC spheroids. To evaluate the stiffness of the spheroids, we introduce the stiffness index, which essentially shows a spring constant per unit size of the spheroid at a certain time during measurement. From the measurement results, we confirmed that the stiffness index firstly increased during the days of culture, although after four days of culture, the stiffness index decreased. We confirmed that the proposed system can measure the stiffness of HUVEC/MSC spheroids.

## 1. Introduction

Collective evidence highlights the functional importance of tissue stiffness, not only in organ development and regeneration but also in quality validation and verification of in vitro engineered tissues [1,2]. Above all, multi-cellular spheroids are used as fundamental units for organ building and organ transplantation [3,4,5,6]. In particular, the co-cultured spheroids of human umbilical vein endothelial cells (HUVECs) and mesenchymal stem cells (MSC) have been studied as a 3D culture model and used in the field of bone tissue engineering [7,8]. While HUVEC/MSC spheroids have superior biological characteristics such as promoting vascularization and high cell viability, they also have a unique mechanical characteristic, i.e., their size decreases after self-aggregation during culture [9]. In relation to the aggregation of cells, mono-cultured HUVECs do not self aggregate. In contrast, mono-cultured MSC aggregates and condenses during culture [9,10,11]. As a result, the size of MSC spheroids decreases during culture. It is considered that the decrease in size is related to the contractility caused by actomyosin, and Rho-associated kinase (ROCK) contributes to regulating the contractility of the actomyosin [12,13]. By inhibiting the ROCK of the MSC spheroid, the size decreasing ratio and cell density are lower than those of non-treated MSC spheroids [14]. Therefore, it can be considered that the decrease in size of HUVEC/MSC spheroids is mainly caused by the contractility generated by the actomyosin of MSCs, which are intracellular frames [4,9].

Herein, under the assumption that the size transition of HUVEC/MSC spheroids is caused by a change in the intracellular frames, we hypothesize that there are temporal transitions in the mechanical characteristics during culture; to date, this has not been well investigated. Against this background, we herein focus on the mechanical characterization of HUVEC/MSC spheroids. By measuring each group of spheroids on different culture days, we can expect a temporal transition in mechanical characteristics. In conventional studies, aspiration techniques using a micropipette are commonly used for mechanical characterization of spheroids [15,16,17]. It takes more than a 100 min to measure a single spheroid. Moreover, when the micropipette moves to the target point, disturbance flow occurs, so it is difficult to reduce the time interval of measurement. On the other hand, methods using a microfluidic chip have been proposed for mechanical characterization of single cells [18,19,20,21,22,23]. These methods deliver the target cells to the measurement point by flow and they can reduce the time interval of measurement. As for the mechanical characterization methods using microfluidic chip, we can categorize them into two types. One is indirect mechanical characterization type, and the other is the direct mechanical characterization type. The former type measure the deformability from deformation amount of target under deforming cells by pinched microchannel or hydrodynamic force [18,19,20,21]. This type can measure in a high-throughput. However, measurement accuracy of cells highly depends on flow condition because input force relates flow velocity and cell size. As a result, this method is difficult for adjusting the spheroids whose sizes change with culture state. The latter type measures the stiffness from deformation amount and reaction force of target cells with high accuracy by combining the force sensor and on-chip probe into the microfluidic chip [22,23]. Combining with displacement reduction mechanisms for magnetic actuation, the on-chip probe is actuated with high-resolution. Although it is required to actuate the on-chip probe with long strokes for measuring the size changing samples, this actuation method is difficult for achieving long stroke actuation for the measurement of the spheroids whose sizes change with culture state due to the displacement reduction mechanism. In order to measure the size changing samples using the microfluidic chip, we propose a whole chip deformation mechanism as an actuation method for the force sensor probes. In addition, for evaluation of the mechanical characteristics of the spheroid, which generally have nonlinearity in the deformation–force relationship, we introduce the stiffness index (*SI*). By using the constructed measurement system, we evaluated the *SI* of HUVEC/MSC spheroids. Through the experiments, we found that the *SI* changed with days of culture.

## 2. Materials and Methods

### 2.1. Mechanical Characterization Using a Microfluidic Chip with Force Sensor Probes

**Figure 1:**
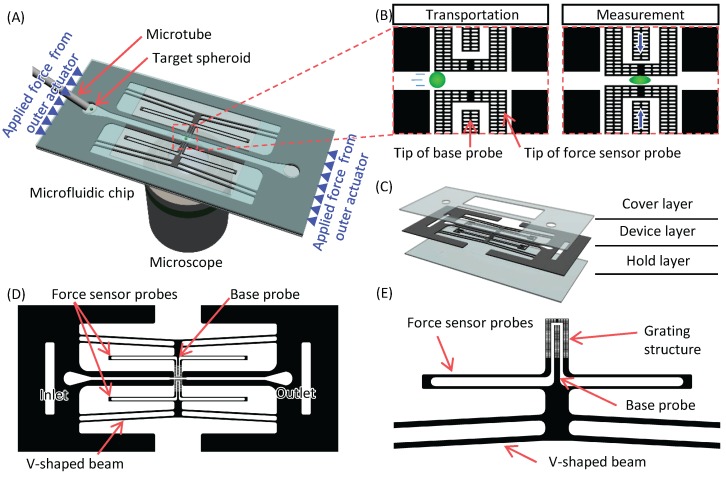
Conceptual images of mechanical characterization system. (**A**) A mechanical characterization system combined with a microfluidic system; (**B**) sequence of measurement; (**C**) components of the chip; (**D**) details of the device layer; and (**E**) magnified view of the V-shaped beam and force sensor probe.

Figure 1 shows conceptual images of the mechanical characterization system. The measurement system is based on a microfluidic system using a chip, as shown in Figure 1A. The chip contains two force sensor probes as part of the microchannel at the measurement point. The target spheroids are introduced into the microchannel and transported to the measurement point of the chip via a microtube using the flow generated by a syringe pump. A position of the target spheroid is manually controlled by a syringe pump based on images taken through a charge coupled device (CCD) camera attached to a microscope. Force sensor probes are simultaneously actuated when the target spheroids are flown at the measurement point. The probes make the target spheroid deform at the center of the microchannel, and its reaction force is measured, as shown in Figure 1B. The chip consists of three layers, a device layer and two glass layers, as shown in Figure 1C. Force sensor probes are fabricated in the device layer. Since HUVEC/MSC spheroids shrink during culture, it is necessary to actuate the force sensor probe with a long stroke. In order to achieve simultaneous actuation with a long stroke, we propose a whole chip deformation mechanism as an actuation method of force sensor probe. Figure 1D shows the details of the chip design. Each force sensor probe is connected to a V-shaped beam as shown in Figure 1E. Details of whole chip deformation mechanism are shown as follows: when the chip is compressed by the actuators beside it, the V-shaped beams transmit a displacement in a horizontal direction to the displacement in a vertical direction. Since the V-shaped beam works as a displacement magnification mechanism, we can actuate the force sensor probes with a long stroke. When a high-resolution outer actuator is utilized in the system, we can expect a long stroke with high-resolution. The force sensor probe contains a beam type force sensor, and the reaction force is calculated from the deformation amount of the sensor beam. The deformation amount of the sensor beam is measured by the image sensor attached to the microscope as the distance between the tip of the base probe, which is connected to the V-shaped beam, and the tip of force sensor probe. The measurement accuracy of the reaction force and the deformation of the spheroids depend on the image resolution. A higher magnification lens is needed; however, there is a magnification limit. In order to measure displacement with high accuracy, we used a sampling moiré method. In order to measure displacement with high accuracy, we used a sampling moiré method [24,25]. By measuring phase shifts that are generated by the grating structure and array architecture of the image sensor of the CCD camera, we can obtain a higher displacement accuracy than the original resolution of the image, which corresponds to one pixel. Therefore, the grating structures are fabricated in the tip of force sensor probes and the tip of base probes.

### 2.2. Design of V-Shaped Beam and Force Sensor Probe

**Figure 2:**
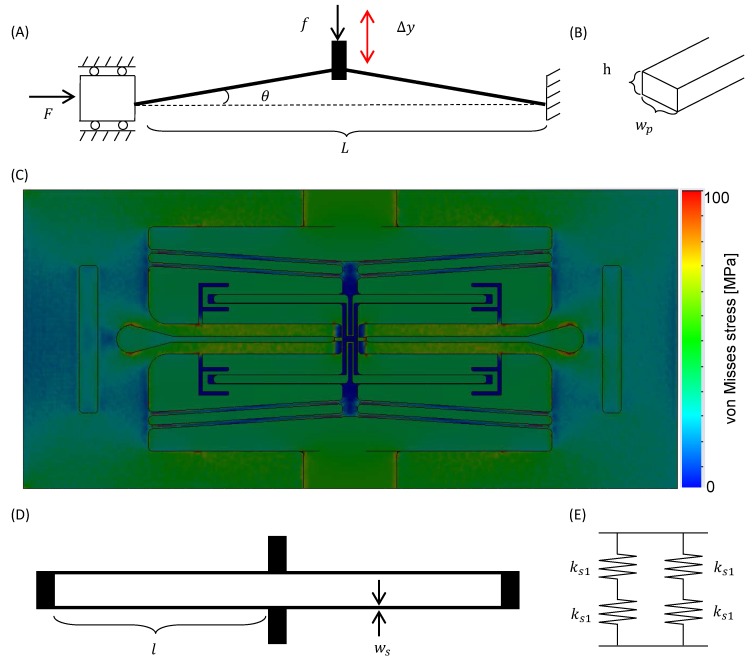
Theoretical model of the V-shaped beam. (**A**) Model configuration of the V-shaped beam; (**B**) cross section of the V-shaped beam; (**C**) finite element method (FEM) image of the von Mises stresses; (**D**) model configuration of the force sensor probe; (**E**) mechanical model of the force sensor probe.

For measuring the mechanical characteristics of spheroids, long stroke actuation of the V-shaped beam such as 100 μm is required with high-resolution. Furthermore, sufficient output force to push the spheroids is also needed. Figure 2A shows the theoretical model of a V-shaped beam, where one end is fixed and the other end is guided along the horizontal axis, in the same manner as in [26,27]. Figure 2B shows the cross-section of the beam. The displacement of the V-shaped beam Δy is expressed by

(1) $$\Delta y=\frac{2}{k_{v}}\left(\tan\theta -\frac{f}{2F}\right)\tan\frac{k_{v}L}{4}+\frac{fL}{4F}-\frac{L}{2}\tan\theta ,$$

where

$$k_{v}=\sqrt[{}]{\frac{F}{EI_{p}}},I_{p}=\frac{hw^{\prime 3}}{12},w^{\prime }=\frac{w_{p}}{\sqrt[{3}]{2}}.$$

The meaning of the parameters in these equations is explained in Table 1. In this study, we set *E* as 169 GPa, which is the Young’s modulus of Si. We designed the V-shaped beam to achieve a displacement Δy that is longer than 100 μm by considering the size of a spheroid of less than 200 μm. In addition, the V-shaped beam must be rigid enough to allow the undesirable displacement due to the push-back force of the deformed spheroid to be neglected. From these conditions, we set *L* = 20 mm, θ = 3°, *h* = 200 μm, and $w_{p}$ = 200 μm. By using the finite element method (FEM) analysis on the whole chip, we evaluated the designed V-shaped beam, and achieved a displacement Δy of 100 μm when the input force F=310 N was applied. Figure 2C shows the von Mises stress map of the FEM analysis in this condition including the cover layer but not displayed (see also Supplementary Materials Videos S1 and S2). The blue area shows the area where the von Mises stress is 0 MPa, and the red area shows the area where the von Mises stress is more than 100 MPa. When 100 μN is applied to the tip of force sensor probe as the reaction force of spheroid *f*, which is higher than the expected reaction force, the V-shaped beam was pushed back 13 nm in the FEM analysis. This displacement is much smaller than the measurement accuracy of displacement; therefore, in this condition, the output force of the V-shaped beam is much higher than the reaction force of the spheroid.

The force sensor probe contains beam-type force sensors, and the reaction force of the deformed spheroid is measured from the deformation of the sensor beam. We apply a folded spring to the force sensor beams. Figure 2D shows a theoretical model of the force sensor probe. The folded spring is regarded as four small springs whose spring constant is represented as $k_{s1}$. Two springs are serially connected as a unit, and the unit is parallelly arranged as shown in Figure 2E. In this case, $k_{s1}$ is expressed by

(2) $$k_{s1}=\frac{12EI_{s}}{l^{3}},I_{s}=\frac{hw_{s}^{3}}{12},$$

where $I_{S}$ is the second moment of the area of the force sensor beam, *l* is the length of force sensor beam, and $w_{s}$ is the width of the force sensor. Finally, the spring constant of the total folded spring $k_{s}$ is expressed by

(3) $$k_{s}=k_{s1}=\frac{Ehw_{s}^{3}}{l^{3}}.$$

Eventually, by considering the fabrication processes of the chip, we set *l* = 6.5 mm, and $w_{s}$ = 15 μm, respectively, and $k_{s}$ is calculated at 0.42 N/m.

### 2.3. Fabrication Processes

**Figure 3:**
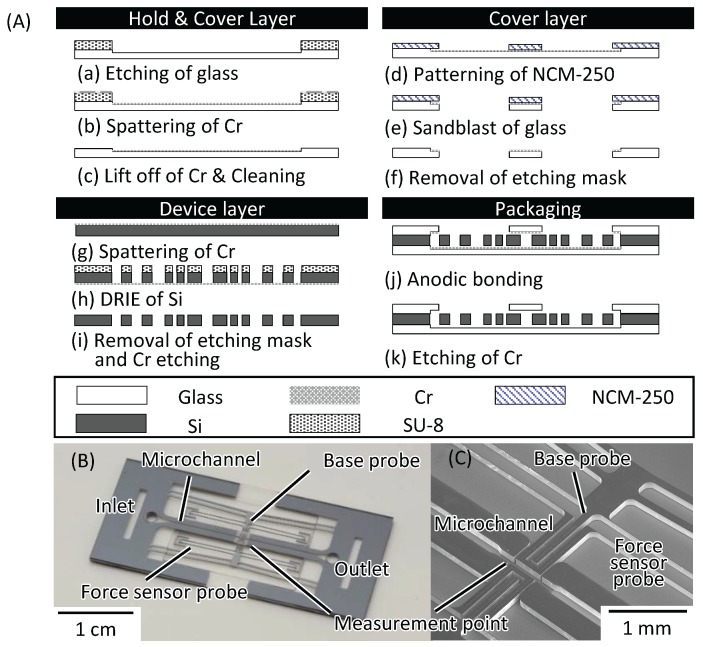
Fabrication of the chip. (**A**) Fabrication process; (**B**) Photograph of the fabricated chip; (**C**) Scanning electron microscopy (SEM) image of the measurement point.

Figure 3A shows the details of the fabrication process of the chip. To actuate the base probe and force sensor probe without friction between them, there is a 15 μm gap between them.

(a) After piranha cleaning of borosilicate glass (TEMPAX Float, DAICO MFG CO. LTD., Kyoto, Japan), PMER (LA900TM, TOKYO OHKA KOGYO CO. LTD., Kanagawa, Japan) was patterned as an etching mask. Then, the glass layer was etched using an inductively coupled plasma (ICP) etcher (RIE-800iPB, SAMCO INC., Kyoto, Japan).
(b) A Cr layer was spattered to prevent the base probes and force sensor probes from bonding to the glass layer.
(c) The Cr layer was lifted off and the glass layer was cleaned using piranha solution.
(d) NCM250 (Nikko-Materials Co., Ltd, Tokyo, Japan) was patterned as an etching mask for sandblasting.
(e) The cover layer was sandblasted to fabricate an inlet and an outlet.
(f) The cover layer was cleaned using piranha solution.
(g) In order to prevent over-etching of the Si, Cr was spattered on the Si layer.
(h) SU-8 (SU-8 3010, MicroChem Corp., MA, USA) was patterned as an etching mask. After patterning, the Si layer was etched using ICP etching (Multiplex-ASE-LS, SPP Technologies Co., Ltd., Tokyo, Japan).
(i) The Si layer was cleaned using piranha solution.
(j) Each layer was bonded using an anodic bonding technique.
(k) After bonding of each layer, Cr was etched.

Figure 3B,C show the fabricated chip and a scanning electron microscopy (SEM) image of the measurement point, respectively.

### 2.4. System Configurations

**Figure 4:**
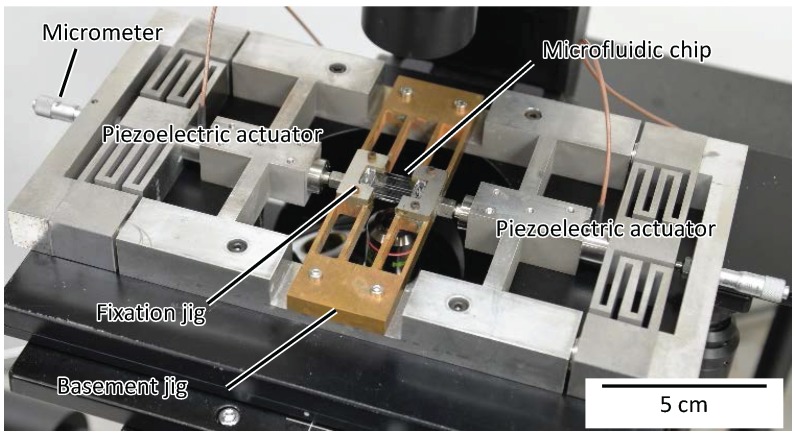
A photograph of the experimental system setup.

Figure 4 shows the experimental system setup. The chip was placed on a basement jig. Two piezoelectric actuators (MPA-UB3, MESS-TEK Co. Ltd., Saitama, Japan) were utilized as outer actuators and were connected to the chip through the basement jig. To adjust the position of the piezoelectric actuators, micrometers (micrometer head MHT4-6.5C, Mitutoyo, Kawasaki, Japan) were connected to the other side of the actuators. A control signal of the piezoelectric actuator was generated by a digital to analog (D/A) board (PEX-340416, Interface, Inc., Atlanta, GA, USA) mounted on a PC. By applying the control signal through the amplifier (MESS-TEK, M-26110-1), we controlled the displacement of the actuator. The displacements of the tip of the base probe and the tip of the force sensor probe were measured by a CCD camera (FL3-U3-13Y3M-C, Point Grey Research, Richmond, BC, Canada) through an objective lens.

### 2.5. Evaluation of Measurement System

**Figure 5:**
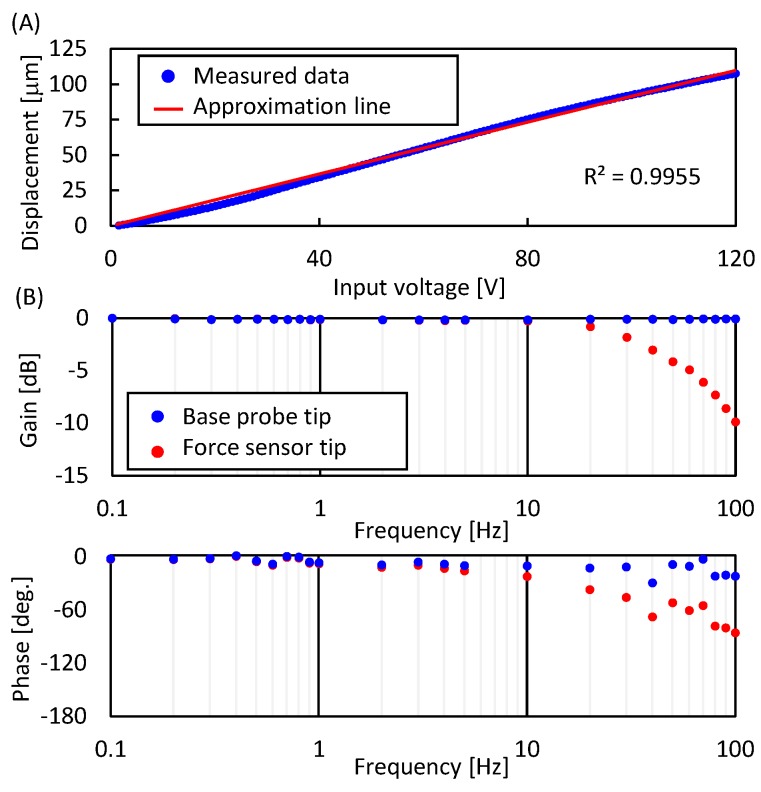
Drive performance of the force sensor probe. (**A**) Relationship between input voltage and displacement of V-shaped beam; (**B**) frequency response of the base probe and the force sensor probe. Sine waves were applied to the piezoelectric actuators, where the applied voltage was 120 V.

The drive performance of the force sensor probe was evaluated in water, as shown in Figure 5. In the following experiments, we used the sampling moiré method to measure the displacement of the probes. The measurement accuracy of the displacement was evaluated in the same manner as in [24], and the result was 27.9 nm (three times the standard deviation). Figure 5A shows the relationship between the input voltage to the piezoelectric actuators and the displacement of the tip of the force sensor probe. The input voltage was increased from 0 to 120 V in 0.3 V steps. The experiments were conducted in triplicate. From Figure 5A, the standard deviations at each voltage were less than 0.1 μm, and the maximum displacement was 108.4 μm as the average value when the input voltage was 120 V. The R-square value of the least squares approximation was 0.998. Since we actuate two probes simultaneously, the actuation stroke is large enough to deform a target whose size is less than 200 μm. From these results, we confirmed that a long actuation stroke was achieved with high-resolution when using the proposed whole chip deformation mechanism. Figure 5B shows the frequency response of the tip of the base probe and the tip of force sensor probe. The gain of the tip of base probe did not decrease up to 100 Hz. However, the gain of the tip of force sensor probe gradually decreased from 10 Hz. Moreover, a phase lag between the tip of the base probe and the tip of the force sensor probe occurred from 2 Hz. From these results, to measure the mechanical characterization of spheroids, we set the ramp input range from 0 to 120 V and the slope of the raising voltage to 60 volt per second. This setting corresponds to approximately 0.5 Hz, and 50 μm per second was expected as the pushing speed.

### 2.6. Sample Preparation

In this study, we used HUVECs (Lonza, Basel, Switzerland) and human MSC (Lonza). In order to fabricate HUVEC/MSC spheroids in vitro, 3.5 × 10^5^ HUVECs and 5.0 × 10^4^ human MSCs were re-suspended in a 24-well spheroid formation plate (Elplasia RB 500 400 NA, KURARAY, Osaka, Japan) with culture medium (Lonza) based on the conventional studies [3,28] and preliminary experiments. HUVEC/MSC spheroids were cultured at 37 °C in a humidified 5 % CO_2_ incubator. ROCK10 spheroids and ROCK100 spheroids were cultured with 10 nM ROCK inhibitor and 100 nM ROCK inhibitor in the culture medium, respectively. Culture time of each kind of spheroid with Day 1, Day 2, Day 3, Day 4, Day 5, Day 6, and Day 7 are over 24 h, 48 h, 72 h, 96 h, 120 h, 144 h, 168 h, respectively.

### 2.7. Gene Expression Analysis

For measuring the gene expression of YAP1, quantitative polymerase chain reaction (Q-PCR) was used. Total RNA were extracted from spheroids using PureLink^®^ RNA Mini Kit (ThermoFisher, Waltham, MA, USA). Complementary DNA was synthesized using a High Capacity cDNA. A reverse Transcription Kit (Applied Biosystems, Foster, CA, USA) was used as a template for PCR amplification. Q-PCR was performed using a Universal probe library system on LightCycler (Roche, Basel, Switzerland) following the manufacturers’ protocols. Expression data were normalized to these for 18S eukaryotic ribosomal RNA. The primer sequences used were YAP1 FW: gacatcttctggtcagagatacttctt YAP1 RV: ggggctgtgacgttcatc use probe #56 (Roche).

## 3. Experimental Results

### 3.1. Reaction Force Measurement

**Figure 6:**
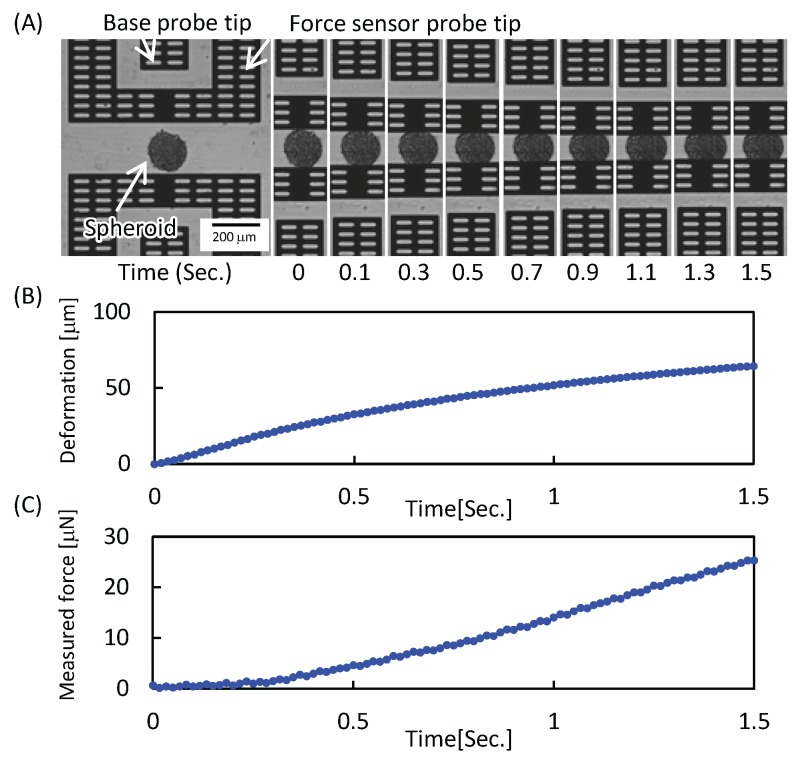
Example mechanical characterization of a human umbilical vein endothelial cells/mesenchymal stem cells (HUVEC/MSC) spheroid. (**A**) Series of photographs during measurement; (**B**) obtained deformation-time curve; (**C**) obtained force-time curve.

In order to evaluate the transition of the mechanical characteristics of the HUVEC/MSC spheroid during culture, the spheroid was measured. In order to avoid the effect of mechanical stimulus, the spheroid measured for its mechanical characteristics was eliminated from the sample group. Figure 6A shows an example of the mechanical characterization results of the HUVEC/MSC spheroid (see also Supplementary Materials Video S3). The target spheroid was deformed by the force sensor probes, after it was positioned at the measurement point of the chip. From Figure 6A, we can see that the distance between the tip of the base probe and the tip of the force sensor probe decreased with respect to the pushing distance. By using these images, we can obtain the deformation-time curve and force-time curve, as shown in Figure 6B,C, respectively. From Figure 6B,C, we can see that the reaction force of the spheroid increased with respect to its deformation.

We would note that the throughput of the measurement was experimentally measured as 92 spheroids per hour, which was an average of 1552 samples. This measurement throughput contributed to the evaluation of the transition of mechanical characteristics.

### 3.2. Introduction of Stiffness Index (SI)

**Figure 7:**
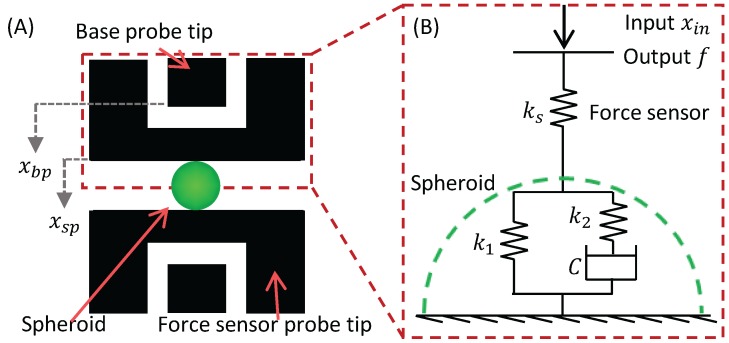
Conceptual diagram of the mechanical characterization of spheroids. (**A**) Definition of $x_{bp}$ and $x_{sp}$; (**B**) theoretical model for *SI* measurement.

In order to evaluate the mechanical characteristics of a spheroid based on the relationship between deformation and the reaction force, which has nonlinearity, we introduce *SI*, which shows the stiffness of the spheroid. We assume a mechanical model for the measurement, as shown in Figure 7. The reactive force of a deformed spheroid can be measured from the displacement of the force sensor probe, as shown in Figure 7A, and displacement between the tip of the base probe and the tip of the force sensor probe $\Delta x_{s}$ is determined by

(4) $$\Delta x_{s}=\Delta x_{bp}-\Delta x_{sp},$$

where $\Delta x_{bp}$ and $\Delta x_{sp}$ are the displacement of the tip of the base probe and the tip of the force sensor probe, respectively. Therefore, the reaction force of a spheroid *f* can be written as:

(5) $$f=k_{s}\Delta x_{s},$$

where $k_{s}$ is the spring constant of the sensor beam. We assume that the spheroid has viscoelastic properties and that the mechanical model can be expressed by a standard linear solid model, which combines the Kelvin–Voigt model and the Maxwell model for the spheroid, as shown in Figure 7B. $k_{1}$ and $k_{2}$ are spring constants of this model, and *C* is viscosity of the dashpot component. In this case, the deformation of the spheroid can be written as $2\Delta x_{s}$ by assuming a symmetric shape. When we give the deformation $x_{in}$ to the base probe, the reaction force *f* is considered as the output of the model. Therefore, the transfer function of the model G(s) including the force sensor is expressed by

(6) $$G\left(s\right)=\frac{k_{s}\left\{k_{1}+\tau s\left(k_{1}+k_{2}\right)\right\}}{k_{s}+k_{1}+\tau s\left(k_{s}+k_{1}+k_{2}\right)},\tau =\frac{C}{k_{2}},$$

where $k_{1}$ and $k_{2}$ are the spring constants of the spheroid, *C* is the viscosity of the spheroid, and *τ* is the time constant of the model. Thus, when we give a ramp command $x_{in}=At$ as an input, the reaction force *f*, which is a function of time, is expressed as

(7) $$f\left(t\right)=A\frac{k_{s}}{k_{s}+k_{1}}\left[tk_{1}+\frac{k_{s}k_{2}\tau }{k_{s}+k_{1}+k_{2}}\left\{1-exp\left(-\frac{t}{\tau }\frac{k_{s}+k_{1}}{k_{s}+k_{1}+k_{2}}\right)\right\}\right],$$

where *A* and *t* are constants that show the pushing speed of the base probe and time, respectively. Spheroid deformation is expressed as

(8) $$x\left(t\right)=x_{in}-x_{s}=At-\frac{f(t)}{k_{s}}.$$

When the deformation-time curve and the force-time curve are linearized in a certain section, we can consider the slope of the line as the index that shows the stiffness of the spheroid. The SI is expressed by

(9) $$SI=\frac{\Delta f}{\Delta x}\times \frac{1}{D},$$

where Δf and Δx are the variation of the reaction force and deformation in a certain section, respectively, and *D* is the equivalent diameter of the undeformed target spheroid. When we put a ramp input $x_{in}$ to the base probe, SI becomes

(10) $$SI=\frac{k_{1}\Delta t+\frac{k_{s}k_{2}\tau }{k_{s}+k_{1}+k_{2}}exp\left(-\frac{t}{\tau }\frac{k_{s}+k_{1}}{k_{s}+k_{1}+k_{2}}\right)\left\{1-exp\left(-\frac{\Delta t}{\tau }\frac{k_{s}+k_{1}}{k_{s}+k_{1}+k_{2}}\right)\right\}}{\Delta t-\frac{k_{2}\tau }{{\left(k_{s}+k_{1}+k_{2}\right)}^{2}}exp\left(-\frac{t}{\tau }\frac{k_{s}+k_{1}}{k_{s}+k_{1}+k_{2}}\right)\left\{1-exp\left(-\frac{\Delta t}{\tau }\frac{k_{s}+k_{1}}{k_{s}+k_{1}+k_{2}}\right)\right\}}\times \frac{1}{D}.$$

From Equation (8), *SI* depends on time *t* and time range Δt for the measurement. In other words, we can evaluate the stiffness of the spheroid from the deformation-time curve and the force-time curve. The value of *SI* is essentially a spring constant per unit size of spheroid at a certain time during measurement. We would note that, because *SI* also depends on $k_{s}$, the force sensor that has a fixed spring constant should be used for comparing the *SI* values of different spheroid groups. In addition, in the following sections, we apply Ramanujan’s second approximation [29] to calculate the diameter of a spheroid because it is not a perfect sphere.

### 3.3. Mechanical Characterization

**Figure 8:**
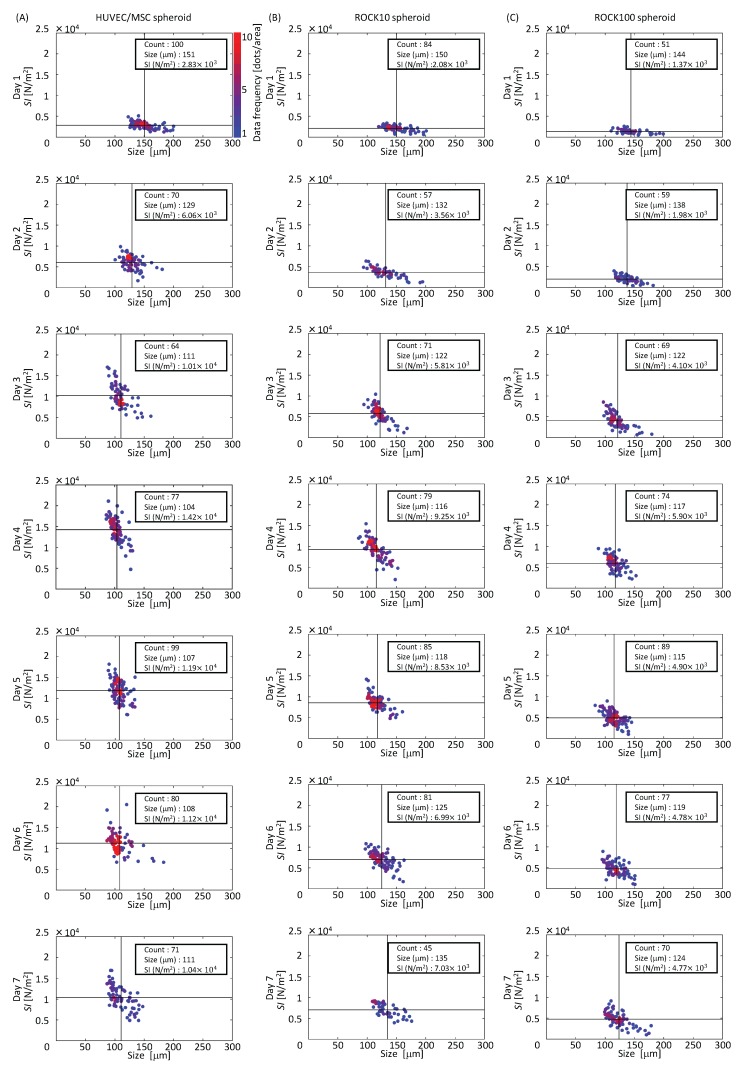
Transitions of measured *SI* and size of spheroids. (**A**) Maps of HUVEC/MSC spheroids; (**B**) maps of Rho-associated kinase (ROCK) 10 spheroids; (**C**) maps of ROCK100 spheroids.

We evaluated the transition of mechanical characteristics using the *SI* that was calculated from measured data in the time *t* = 0.5 (s) and time range Δt=0.2 for all samples. Figure 8A shows the maps of the diameter and *SI* of HUVEC/MSC spheroids on different culture days. In these figures, color indicates the frequency of the data in an area divided into 8-by-8. Note that the red dots mean that there are more than nine dots in an area. In addition, the vertical and horizontal black lines show the average size and *SI* of the spheroid group, respectively. Initially, the average *SI* increased with culture days; however, it decreased after day 4. According to the Student’s *t*-test (MATLAB R2016a, MathWorks, Natick, MA, USA), there was a significant difference between day 1 and the other culture days. Every *p*-value for the comparison between day 1 and the other culture days was much lower than 0.001.

## 4. Discussion

In order to evaluate the effect of myosin on the stiffness, HUVEC/MSC spheroids cultured with ROCK inhibitor 10 nM (ROCK10 spheroid) and 100 nM (ROCK100 spheroid) were also measured. Figure 8B,C shows the maps of the diameter and *SI* of ROCK10 and ROCK100 spheroids at different culture days. From these maps, we can see that the transition of *SI* in ROCK10 and ROCK100 showed a similar tendency to *SI* in the HUVEC/MSC spheroids. Moreover, the *SI* of the HUVEC/MSC spheroids were higher than those of ROCK10 spheroids and ROCK100 spheroids on each day. There were also significant differences between HUVEC/MSC and ROCK10, HUVEC/MSC and ROCK100, and ROCK10 and ROCK100. Every *p*-value for the comparison between day 1 and the other culture days was much lower than 0.001. These results indicate that the activity of myosin is one factor that affects the *SI*.

**Figure 9:**
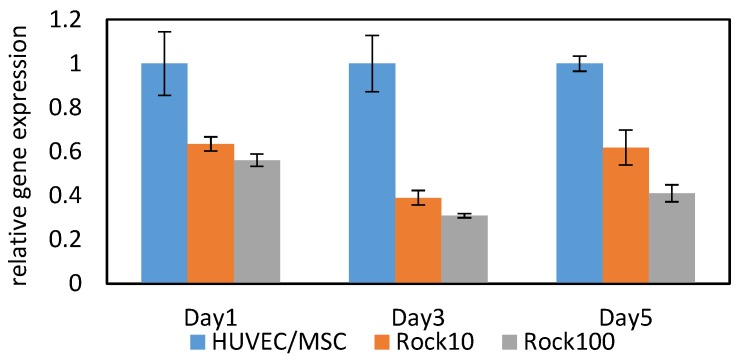
Comparison of relative gene expression of YAP1 in HUVEC/MSC, ROCK10 and ROCK100 spheroids.

We evaluated YAP1 gene expression in HUVEC/MSC, ROCK10 spheroids, and ROCK100 spheroids. YAP1 is a member of the Hippo signalling pathway. Although there is no direct evidence connecting YAP1 and the Rho signaling pathway, it is considered that the activities of actin and myosin are functionally connected to the activity of YAP/TAZ in stem cells such as MSC [30,31]. Moreover, it is revealed that the expression amount of YAP1 correlates with the stiffness of tissue [17]. From this understanding, we measured the gene expression of YAP1 in HUVEC/MSC, ROCK10, and ROCK100 spheroids. Figure 9 shows the results of the Q-PCR. Gene expression of YAP1 was normalized with respect to the gene expression of HUVEC/MSC. Error bars show the standard deviations, which are calculated from duplicate measurements in Q-PCR. From these results, we can see that the gene expression of HUVEC/MSC spheroids on all culture days was higher than those of ROCK10 and ROCK100 spheroids. Moreover, the gene expression of ROCK10 spheroids on all culture days was higher than that of ROCK100 spheroids. These results indicate a similar tendency to the measured *SI*. The correlation factors between SI and YAP1 in HUVEC/MSC, ROCK10 and ROCK100 spheroids ware 0.74, 0.87, and 0.85, respectively. From these results, we confirmed that the transition of *SI* has a similar tendency to the YAP1 gene expression.

## 5. Conclusions

In this study, we measured the transition of the mechanical characteristics of HUVEC/MSC spheroids during culture using a microfluidic chip that was integrated with force sensor probes. By combining the microfluidic system with the measurement system, measurement throughput was achieved up to 92 spheroids per hour. In order to evaluate the stiffness of spheroids that have nonlinearity in the deformation-time curves and force-time curves, the *SI* was introduced. Then, we measured the *SI* of HUVEC/MSC, ROCK10, and ROCK100 spheroids. The results indicate that there was a relationship between the mechanical characteristics and the activity of myosin. Since *SI* showed a similar tendency to YAP1 gene expression, the mechanical characterization method using the microfluidic chip will be less invasive and faster for evaluating the spheroids.

## Figures and Tables

**Table 1:** Parameters for design of the V-shaped beam.

Parameter	Meaning
*F*	Input force
*f*	Reaction force of the spheroid
*L*	Length of V-shaped beam
θ	Elevation angle of V-shaped beam
*E*	Young’s modulus of Si
$I_{p}$	Second moment of area of V-shaped beam
$w^{\prime }$	Equivalent parallel probe width of V-shaped beam
$w_{p}$	Probe width of V-shaped beam

## Supplementary Materials

The following are available online at www.mdpi.com/2072-666X/7/12/221/s1, Video S1: FEM analysis of von Mises stress. Video S2: FEM analysis of displacement. Video S3: Example of mechanical characterization.
